# Use of obinutuzumab for refractory autoimmune thrombocytopenia secondary to CLL

**DOI:** 10.1002/jha2.78

**Published:** 2020-08-12

**Authors:** Rohitashva Agrawal, Reinhold Munker, Maxwell M. Krem

**Affiliations:** ^1^ Division of Hematology and Blood & Marrow Transplantation Markey Cancer Center University of Kentucky College of Medicine Lexington Kentucky

## 1 INTRODUCTION

Chronic lymphocytic leukemia/small lymphocytic lymphoma (CLL/SLL) is a common hematologic malignancy that is complicated by frequent secondary autoimmune complications, including hemolytic anemia and immune thrombocytopenic purpura (ITP), occurring in 10‐25% of cases [1, 2]. Pure red cell aplasia and autoimmune granulocytopenia have also been identified. These complications may pose risk of serious morbidity and mortality [3].

First‐line management of ITP in CLL consists of corticosteroids. Secondary immunosuppressive modalities include intravenous immunoglobulin (IVIG), rituximab, and splenectomy [2]. Rituximab is a useful adjunct in therapy of autoimmune cytopenias related to CLL [2, 3]. Splenectomy has high success rates but exacerbates susceptibility to encapsulated organisms and requires prophylactic immunizations. Limited data sets have demonstrated benefit of the thrombopoietin (TPO) receptor agonists romiplostim and eltrombopag, which are FDA approved for ITP that is nonresponsive to steroids, IVIG, or splenectomy [4].

Anti‐CD20 monoclonal antibodies directed against CD20 have the potential to neutralize both autoimmune and malignant B cells. Anti‐CD20 antibody mechanisms of action include antibody‐dependent cell‐mediated cytotoxicity (ADCC), complement‐dependent cytotoxicity (CDC), and apoptosis [5]. Anti‐CD20 antibodies are classified as type 1 (rituximab and ofatumumab) or type 2 (obinutuzumab). Type 1 antibodies organize CD20 molecules into “rafts” and exert their effects primarily by ADCC and CDC, and less by apoptosis. Type 2 antibodies utilize apoptosis to a greater degree and also exert more potent ADCC [6]. The type 1 antibody rituximab has demonstrated efficacy in management of auto‐immune hemolysis and thrombocytopenia due to CLL, including patients who have not responded to other interventions [2, 3]. We present a case in which the type 2 antibody obinutuzumab successfully treated ITP secondary to CLL.

## 2 CASE PRESENTATION

The patient was diagnosed with CLL in January 2012 after presentation with leukocytosis, lymphadenopathy, and drenching night sweats. Peripheral blood flow cytometry was positive for CD5, CD19, CD20 (dim), CD22, CD23, CD43, CD45, and ZAP70. Cytogenetics was not performed at initial diagnosis. She received four cycles of fludarabine, cyclophosphamide, and rituximab (FCR) and derived complete response.

In June 2015, 3.5 years after initial diagnosis, she presented with cough, shortness of breath, and significant bruising, despite receiving monthly IVIG for frequent upper respiratory infections. Platelet count was 4000/mm^3^. She did not have lymphocytosis or anemia. Bone marrow biopsy and aspirate revealed 25% marrow involvement by CLL, suggesting that thrombocytopenia was of autoimmune etiology.

She received prednisone 1 mg/kg for 2 weeks, with no platelet response, followed by prednisone taper. As the next line of management, she received rituximab, given as 4 weekly doses of 375 mg/m^2^. She had platelet response to 83 000/mm^3^; unfortunately, 1 month after initiating rituximab she developed dyspnea and hypoxemia. She was diagnosed with DAH based on chest CT and bronchoscopy with lavage. Her course was complicated by presumed esophageal intubation and gastric perforation, but the patient recovered and was discharged. By the time of discharge, platelet response had reached 364 000/mm^3^.

After approximately 1 year of continued disease remission, she had CLL progression with increasing absolute lymphocyte count. FISH studies showed p53 deletion (del17p) in 87% of cells. Trisomy 12, deletion ATM, deletion 13q, and t(11;14) were negative. She was treated with idelalisib (rituximab omitted due to history of DAH). WBC count responded to therapy. However, after 6 months of idelalisib, she had coughing and dyspnea. CT scan of the chest showed new reticulonodular opacities in the superior segment of the right lower lobe, felt to be consistent with pneumonitis. Idelalisib was discontinued, and symptoms improved.

Shortly after stopping idelalisib, she presented on May 22, 2017 with platelet count of 4000/mm^3^. She had not experienced bleeding but had extensive bruising. She was admitted and received IVIG 1 g/kg on two consecutive days. She had temporary platelet response that lasted 3‐4 weeks. She was admitted again and was given IV methylprednisolone 500 mg x 4 doses. Platelets improved to 141 000/mm^3^. She was discharged with prednisone 20 mg daily. The response lasted approximately 2‐3 weeks before platelet count declined to 14 000/mm^3^ on July 14, 2017 and then 10 000/mm^3^ on July 19, 2017.

**FIGURE 1:**
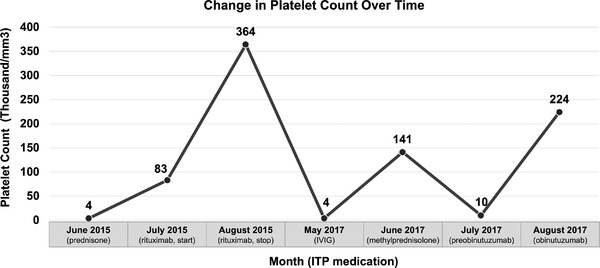
Changes in platelet count (*y* axis) over time (*x* axis) in response to medications

The patient began treatment with obinutuzumab as a single agent on July 19, 2017, with the first 1000 mg dose split over 2 days, with the intent of suppressing ITP and providing control of her CLL. Platelet count immediately jumped from 10 000 to 87 000/mm^3^ and then to 224 000/mm^3^ in 1 week (Figure 1). Cycle 1 was complicated by pneumonia, requiring hospital admission. Cycle 5 was complicated by neutropenia with ANC 100. She was treated with tbo‐filgrastim, which normalized her neutrophil count. She received six cycles in total and had no additional serious adverse events. As of May 2020, the patient had received subsequent therapy with ibrutinib, venetoclax, and acalabrutinib. Although we are not certain of the duration of platelet response due to subsequent treatment, there had been no recurrence of ITP.

## 3 DISCUSSION

Our patient's case was notable for a history of brief or no response to steroids on three occasions, including high‐dose methylprednisolone. She also received high‐dose IVIG, which only yielded a brief response. Her secondary ITP recurred despite evidence of remission on therapy with idelalisib. She had a prior excellent response to rituximab that was unfortunately suspected to be complicated by the life‐threatening adverse event of DAH, and further rituximab was thought to be contraindicated [7, 8]. We wished to obtain a prolonged platelet response, as experienced with rituximab, but with less risk to the patient. Therefore, we turned to the second‐generation anti‐CD20 monoclonal antibody obinutuzumab.

ITP patients may experience life‐threatening complications due to low platelet counts [3]. ITP requires urgent and effective control. We believed that obinituzumab might yield response kinetics similar to those seen previously with rituximab. Obinutuzumab is a novel type 2 anti‐CD20 monoclonal antibody [9]. Possible explanations for efficacy in an autoimmune condition[10] and avoidance of the pathophysiologic process that led to DAH include its fully humanized sequence or its different epitope specificity from rituximab. We also anticipated that obinutuzumab would provide disease control [11], which has also been shown in the single‐agent treatment setting [12]. Obinutuzumab induced a dramatic platelet response, which we attribute primarily to its immunosuppressive activity. Its action on the primary disease may have helped control the magnitude and the root cause of ITP.

Other emerging therapies for refractory ITP in CLL include eltrombopag[4]; romiplostim[13]; rituximab, cyclophosphamide, and dexamethasone (RCD) [14]; and alemtuzumab [3]. We deferred splenectomy [4] to avoid further immune impairment.

To the best of our knowledge, this is the first report of obinutuzumab's efficacy in either primary or secondary ITP. We believe that our case demonstrates that obinutuzumab is a viable option for patients with secondary ITP, especially those who cannot receive rituximab, and that prospective study of this agent in patients with secondary ITP is warranted.

## CONFLICT OF INTEREST

The authors declare that there is no conflict of interest.

## CONSENT

The patient has given consent to the inclusion of material pertaining to herself and acknowledges that she is not identifiable via the paper. We have fully anonymized the patient.

## Data Availability

The data that support the findings of this study are available on request from the corresponding author. The data are not publicly available due to privacy or ethical restrictions.
